# Dolphinfish Bycatch in Spanish Mediterranean Large Pelagic Longline Fisheries, 2000–2010

**DOI:** 10.1100/2012/104389

**Published:** 2012-03-12

**Authors:** David Macías, José C. Báez, Salvador García-Barcelona, José M. Ortiz de Urbina

**Affiliations:** Centro Oceanográfico de Málaga, Instituto Español de Oceanografía, Puerto Pesquero Fuengirola, Málaga, 29640 Fuengirola, Spain

## Abstract

The aim of this paper is to describe the dolphinfish bycatch rates in the longline fisheries of the Western Mediterranean and modelling the nominal bycatch abundance and distribution of dolphinfish from the Spanish Mediterranean as a function of technical, geographical, and seasonality factors. Our results indicate that the impact of the pelagic and semipelagic longline on the dolphinfish population is relatively low (1.083 fishes per 1000 hooks), in contrast with the greater effect on the target species population. We obtained a statistically significant logistic model, with the following factors: technical characteristics of the fishery, geographical location, and seasonality. Drifting surface longliners targeting albacore is the gear with the highest effect on Mediterranean dolphinfish population. The technical characteristics of the fishery and seasonality factors have an important role in explaining the absence or presence of dolphinfish bycatch in the different boat strata, gear types, and seasons. Moreover, sea surface temperature and lunar phases also present additional explanations. Lunar phase as SST has been frequently used as an explanatory variable affecting catch rates of dolphinfish.

## 1. Introduction

Incidental catch or bycatch represents 8% of global fisheries production [1]. Bycatch is defined as any unwanted species caught during normal fishing operations and may include nontarget fish species, marine mammals, turtles, sharks, and seabirds [2, 3].

Dolphinfishes (*Coryphaena hippurus *and* Coryphaena equiselis*) are highly migratory pelagic species which inhabit tropical, subtropical, and temperate waters. They constitute a valuable seasonal resource for small-scale fleets. Traditionally, dolphinfish has been an important food resource for the Mediterranean people. The Mediterranean landings of these species have increased regularly in the last decade [4]. Nevertheless, the assessment and management of dolphinfish is difficult mainly due to the scarcity of data on biology, migratory patterns, and exploitation of these species in the Mediterranean.

Dolphinfishes in the Mediterranean support both commercial small-scale fishing and recreational fisheries [5, 6]. In Malta, Tunisia, Sicily, and Balearic Islands from the end of summer to autumn, dolphinfish juveniles are caught using Fish Attracting Devices [7–9]. These species are also caught as a bycatch of commercial longline fisheries [10, 11]. The Western Mediterranean Sea is an important fishing ground where the Spanish drifting longline fishery operates targeting mainly swordfish *Xiphias gladius*, bluefin tuna *Thunnus thynnus*, and albacore *T. alalunga*. In this context, identification of the principal factors that determine this bycatch is basic to improve the assessment and management of the Mediterranean dolphinfish stocks.

The aim of this paper is to describe the dolphinfish bycatch rates in the longline fisheries from Western Mediterranean and modeling the nominal bycatch abundance and distribution of dolphinfish from the Spanish Mediterranean as a function of technical, geographical, and seasonality factors.

## 2. Material and Methods

**Figure 1:**
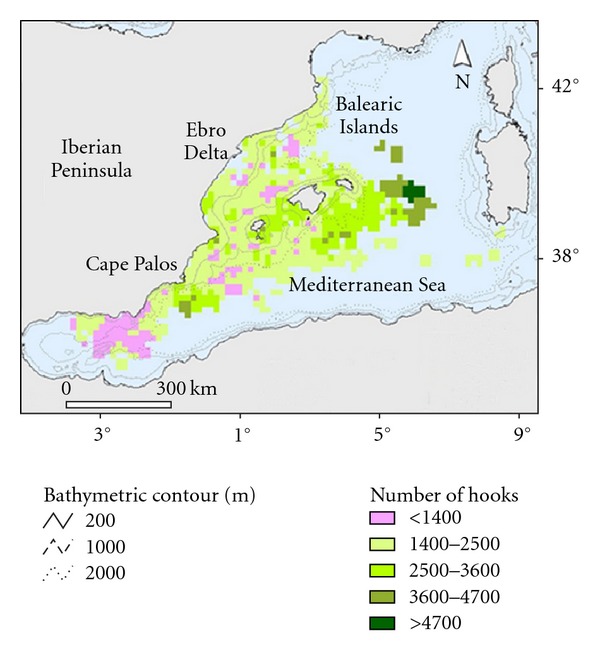
Spatial distribution of observed fishing effort and known fishing grounds.

Catch and effort data for longline fisheries were collected by the Spanish Oceanographic Institute (IEO) on-board observer training program, planned according to International Commission for the Conservation of Atlantic Tunas (ICCAT) recommendations. The positions of the fishing grounds and spatial distribution of gear effort are shown in Figure 1. We classified the fleet into six strata (Table 1), according to differences in target species, operational depth, and technical characteristics (more detailed information in [12]). The positions of the fishing grounds and spatial distribution of gear effort are shown in detail in García-Barcelona et al. [12].

 The IEO on-board observer Program provided commercial fish catch and bycatch data collected on longline vessels from 1997 to 2010. Observers were assigned based on strata. Dolphinfish bycatch data were collected from 2000 to the present day, so only 2000–2010 period is included in the present study. For each fishing set observed, data were recorded on fishing set location, time of setting and hauling; environmental data (sea surface temperature, distance to the coast, depth and weather conditions, moon phase), soaking duration; gear characteristics (total length, mean depth, number of hooks, etc.); type and size of bait; species composition; corresponding biological information (size/weight). Within each set sampled, observers monitored 100% of the total hooks retrieved and recorded information on species composition, number, and estimated weight of both target species and bycatch including dolphinfish. In addition, the environmental variables listed above were also recorded.

With regards to dolphinfish, the objectives of observers were to record captures and identify specimens to the lowest taxonomic level possible. However, at the beginning of the temporal series, as the observers had little experience with dolphinfish, many specimens could not be identified and/or recorded at species level. The accuracy of the data improved gradually reaching and now has a high degree of precision.

*Coryphaena hippurus* and *Coryphaena equiselis* present a very similar appearance, especially in juveniles. For this reason, specific segregation is very difficult. Both species had been recorded in the Mediterranean Sea, but reports of *Coryphaena equiselis* are scarce and its presence in these waters is generally considered as rare. Massutí and Morales-Nin [13] and Carbonell et al. [14] provide some data supporting the uncertainty of the presence of *C. equiselis* in the Mediterranean. For this reason, we considered the *C. hippurus* as unique species bycaught in longline from Mediterranean Sea.

### 2.1. Data Analysis

We calculated annual dolphinfish bycatch rates as the total number of individual dolphinfish caught in a year divided by the number of hooks deployed (CPUE). In addition, we calculated the average annual CPUE as the mean of CPUE per set (all sets in a year) and standard errors for dolphinfish, to explore patterns in the data. A chi-square test [15] was used to test for statistically significant differences in number of dolphinfish caught between gear strata and between levels of fishing effort by year. 

To estimate the average annual dolphinfish bycatch, we calculated the observed annual CPUE (average annual CPUE per set), and then the average number of fish caught each year, extrapolating the observed annual catch rates (CPUE) from the total annual effort. Finally, we calculated the mean number of dolphinfish and standard errors in the period studied. The average annual number of dolphinfish was calculated using the same methodology.

### 2.2. Bycatch Explicative Model

In the first step, as followed in García-Barcelona et al. [12] who followed a hypothetical-deductive method, we performed a binary logistic stepwise forward/backward regression of the presence and absence of dolphinfish bycatch to test whether the probability of incidentally catching a dolphinfish (1 or more) may be forecast by some of these explanatory variables listed in Table 2. With this first step, we standardised the most optimal capture conditions of *Coryphaena sp*. bycatch. This allowed us to delete those sets with structural absences. Many authors recommend the use of logistic regressions for evaluating the effects of environmental conditions and fishing practices on the probability of interactions with bycatches [16–20], and it could relate the probability of an event (e.g., the risk of catching a specimen of *Coryphaena*) with a series of variables and explanatory factors.

By performing a logistic regression of the bycatch presence/absence on each variable separately, we selected a subset of variables significantly related to the distribution of the bycatch. To control for the increase in type I error due to multiple tests (to see [12]), we only accepted those variables that were significant under a False Discovery Rate (FDR) of **q** < 0.05, using the Benjamini and Hochberg procedure (to see [12]). We then performed forward stepwise logistic regression on the subset of significant predictor variables to obtain a multivariate logistic model.

Model coefficients were assessed by means of an omnibus test and the goodness of fit between expected and observed proportions of bycatch events along ten classes of probability values and evaluated using the Hosmer and Lemeshow test (which also follows a Chi-square distribution; low **P**-0.05 would indicate lack of fit of the model) [21]. On the one hand, the Omnibus test examines whether there are significant differences between the −2LL (less than twice the natural logarithm of the likelihood) of the initial step, and the −2LL of the model, using a Chi-squared test with one degree of freedom. On the other hand, the Hosmer and Lemeshow test compares the observed and expected frequencies of each value of the binomial variable according to their probability. In this case, we expected that there are no significant differences for a good model fit.

In addition, the discrimination capacity of the model (trade-off between sensitivity and specificity) was evaluated with the receiving operating characteristic (ROC) curve. Furthermore, the area under the ROC curve (AUC) provides a scalar value representing the expected discrimination capacity of the model. The relative importance of each variable within the model was assessed using the Wald test [21].

In a second step, we created and modelled different probability scenarios according to the qualitative variables that are in the final model. Thus, we modelled for boat strata (LLALB, LLAM, LLHB) between June and November during the study period, the probability of a fishing operation presenting a CPUEw value higher than the average CPUEw for this boat stratum, using binary logistic regression and the variables of Table 3 as explanatory factors. We considered the “Moon effect” as the binary effect of the moon light; consequently, we assigned the value 1 when the moon was right 50% visible, between the waxing gibbous moon-full-moon-last quarter-moon; while we considered 0 when it was less than 50%, between the waning-crescent moon-new moon-waxing crescent moon. The target variable was 1 when the CPUE of a particular set was higher than the mean CPUE for that boat strata pooled together, while we assigned the value 0 when the CPUE was lower than that mean CPUE value.

### 2.3. Spatial Representation of Fishing Area and Effort

Geographical coordinates of all fishing operations (setting and hauling) were recorded using a GPS (Datum WGS 84). The begin set point was used to represent the fishing effort (number of hooks set). Afterwards, effort values were interpolated to grids of 15 × 15 km in order to maintain confidentiality requirements. Dolphinfish bycatch of each set was represented using CPUE (fishes per 1000 hooks). Maps were projected in UTM, zone 31N.

Spatial representations of fishing effort and dolphinfish bycatch were made using ESRI ArcView 3.2 software and the Spatial Analyst and Xtools extensions.

## 3. Results

During the 11 years covered in this study, a total of 2,968 fishing sets were observed, and the number of dolphinfish bycaught was 6,663 fish in 610 positive fishing operations, the average CPUE was 1.08 fishes/1000 hooks, and the CPUEw was 1.82 kg per 1000 hooks.

We classified the fleet into six *strata*, according to differences in target species, operational depth, and technical characteristics (more detailed information in García-Barcelona et al. [12]).

### 3.1. Bycatch Description

All of the six monitored gears in this study caught dolphinfish.  Table 4 shows the average CPUEn per gear and year, and Table 5 shows the average CPUEw per gear and year along the studied period. The mean fork length (FL) for the dolphinfish studied was 62.7 cm.

**Figure 2:**
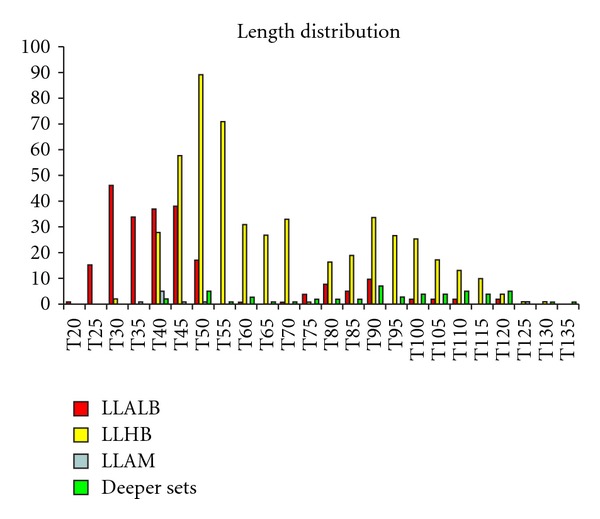
Length distribution per boat strata and gear type. Exist significative differences between boat strata length distribution (chi-squared = 1521.42; *df* = 69; **P** < 0.0001).

Figure 2 shows the length distribution per fleet strata. Length distributions of LLALB (abbreviation as Table 1) and LLHB have a bimodal shape. The first mode for LLALB was 30 cm and for LLHB was 50 cm, the second mode was 90 cm for both fleet strata. There exist significant differences between lengths distributions of all fleet strata studied. The smallest sizes were found in LLALB (average length = 45.6 cm) followed by LLAM (average length = 50.6 cm) and LLHB (average length = 67.6 cm). In general, the greater depth a set was made, the larger were the dolphinfish caught. In this sense, the mean length value of the dolphinfish caught by the deeper sets (made with LLSP, LLPB and LLJAP) was 90.4 cm. Our results also suggest that the smaller hooks tend to capture smaller dolphinfish, while the larger hooks (targeting swordfish and bluefin tuna) tend to select the larger animals. Thus, it is very important to consider gear type when making inferences about the dolphinfish populations based on fisheries bycatch data.

**Figure 3:**
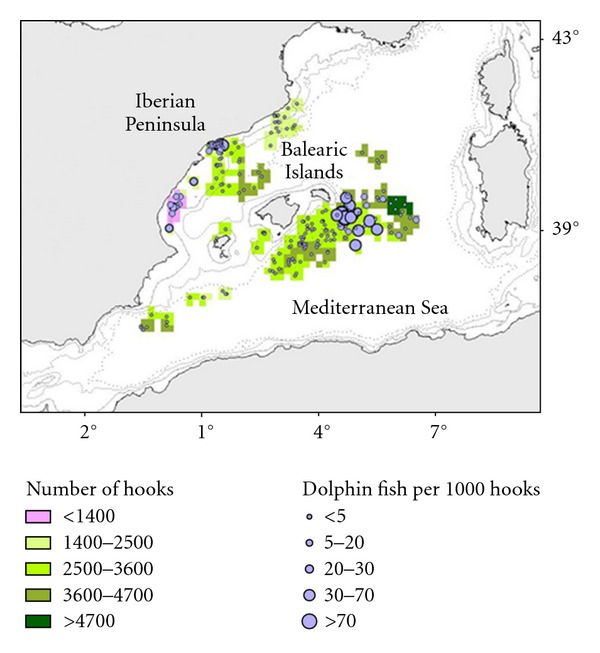
Map of the LLALB fishing ground. Fisheries operation observed and dolphinfish bycatches (number of fishes observed per 1000 hooks) per set.

 LLALB showed a CPUEn of 3.70 fishes per 1000 hooks, the highest CPUEn was recorded in 2006 (9.99 fishes per 1000 hooks) and the lowest in 2000 (0.05 fishes per 1000 hooks). CPUEw show the same trend with the highest catch weight in 2006 and the lowest in 2000. The mean weight of dolphinfish caught by this gear was 0.77 kg.  Figure 3 shows spatial distributions of sets, effort, and its corresponding dolphinfish catch rates for LLALB (CPUEn).

**Figure 4:**
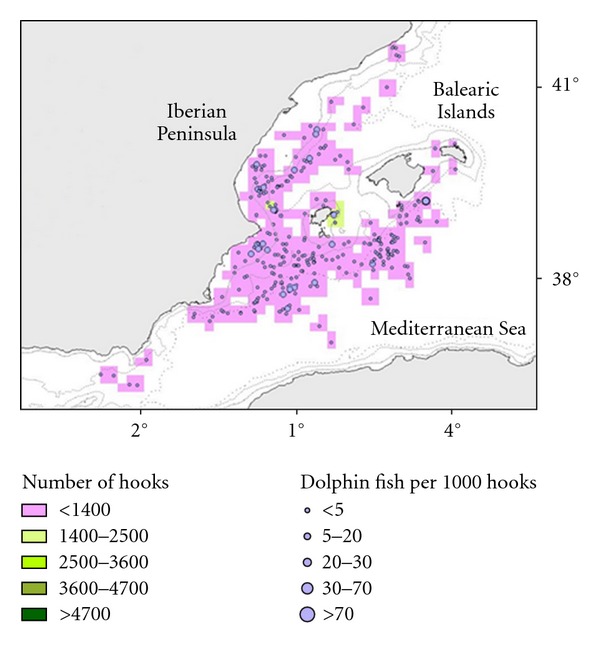
Map of the LLAM fishing ground. Fisheries operation observed and dolphinfish bycatches (number of fishes observed per 1000 hooks) per set.

**Figure 5:**
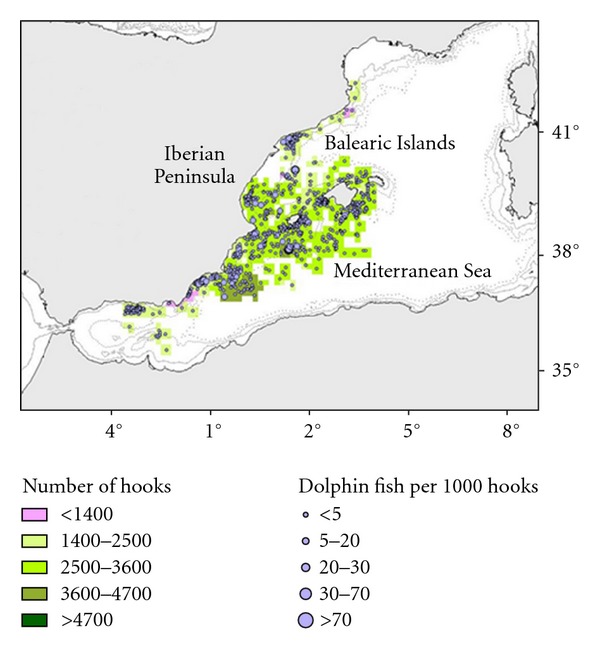
Map of the LLHB fishing ground. Fisheries operation observed and dolphinfish bycatches (number of fishes observed per 1000 hooks) per set.

 LLAM had an average CPUEn of 1.2 fishes per 1000 hooks, lower than that for LLALB. The highest CPUEn and CPUEw were recorded in 2003 (2.29 fishes per 1000 hooks/2.44 kg per 1000 hooks) and the lowest in 2005 and 2009 (0.0 fishes and kg per 1000 hooks). The average weight of dolphinfish bycaught by LLAM was 1.1 kg.  Figure 4 shows observed effort of LLAM and its corresponding dolphinfish catch values. LLHB had an average CPUEn of 0.85, slightly lower than that for LLAM. The highest CPUEn was recorded in 2004 (4.59 fishes per 1000 hooks) and the lowest in 2010 (0.26 fishes per 1000 hooks). CPUEw shows the same trend and the average weight of fishes was 2.7 kg.  Figure 5 shows observed effort of LLHB and its corresponding dolphinfish catch values.

 Regarding spatial distribution of the dolphinfish bycatch, our results indicate that LLALB shows the most heterogeneous catch rates distribution with areas with high catch rates such as the Ebro Delta continental shelf and South East of Menorca Island and areas without catches (Figure 3). LLAM and LLHB show a more homogeneous distribution of catch rates (Figures 4 and 5).

The average annual effort for the Spanish pelagic longline fleet is 13,283,631 ± 1,093,799 hooks (http://www.iccat.es/en/). Based on the average annual effort for the Spanish pelagic longline and the average annual CPUE, an average total bycatch estimate for the fleet for this period was around 14,490 dolphinfish per year, this value corresponding to approximately 24,176 kg per year.

### 3.2. Explicative General Logistic Model

**Figure 6:**
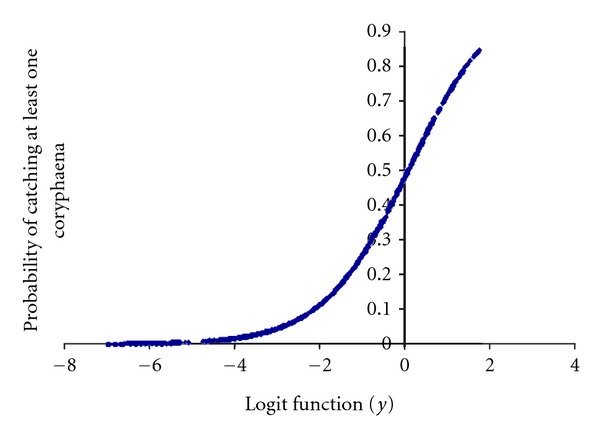
Probability of the incidental capture (1 or more) of a dolphinfish in relation to the binary logistic regression.

We obtained a statistically significant logistic model (Figure 6), with the variables (in order of Wald-value): drifting surface longline targeting albacore (positive relation), October (positive relation), traditional longline targeting swordfish (positive relation), November (positive relation), September (positive relation), American longline targeting swordfish (positive relation), August (positive relation), latitude where the setting started (negative relation), Diurnal (positive relation), March (negative relation), May (positive relation), June (positive relation), April (negative relation), and July (positive relation). The model's goodness of fit was significant according to the Omnibus test (Omnibus test = 907.744, *df* = 14, *P* < 0.001; Hosmer and Lemeshow test = 21.625, *df* = 8, *P* = 0.006). *R* ^2^-Nagalkerke = 0.4, and its discrimination capacity was outstanding (AUC = 0.856).

The logit function (*y*) from logistic regression was

(1) $$\begin{array}{cc} y & =1.905+{\text{LATSS}}^{*}-0.147+\text{LLAM}\{\begin{array}{c} \text{NOT}=-1.282\\ \text{YES}=0 \end{array}\\ & \;+\text{LLHB}\{\begin{array}{c} \text{NOT}=-1.879\\ \text{YES}=0 \end{array}+\text{LLALB}\{\begin{array}{c} \text{NOT}=-2.543\\ \text{YES}=0 \end{array}\\ & \;+\text{DN}\{\begin{array}{c} \text{NOT}=0\\ \text{YES}=0.546 \end{array}+\text{MA}\{\begin{array}{c} \text{NOT}=2.5\\ \text{YES}=0 \end{array}\\ & \;+\text{AB}\{\begin{array}{c} \text{NOT}=2.19\\ \text{YES}=0 \end{array}+\text{MY}\{\begin{array}{c} \text{NOT}=-1.788\\ \text{YES}=0 \end{array}\\ & \;+\text{JN}\{\begin{array}{c} \text{NOT}=-0.742\\ \text{YES}=0 \end{array}+\text{JL}\{\begin{array}{c} \text{NOT}=-0.589\\ \text{YES}=0 \end{array}\\ & \;+\text{AG}\{\begin{array}{c} \text{NOT}=-1.003\\ \text{YES}=0 \end{array}+\text{SE}\{\begin{array}{c} \text{NOT}=-1.971\\ \text{YES}=0 \end{array}\\ & \;+\text{OC}\{\begin{array}{c} \text{NOT}=-3.064\\ \text{YES}=0 \end{array}+\text{NO}\{\begin{array}{c} \text{NOT}=-2.305\\ \text{YES}=0. \end{array} \end{array}$$

Key words: LLHB, traditional longline targeting swordfish; LLAM, American longline targeting swordfish; LLALB, drifting surface longline targeting albacore; LATSS, latitude where the setting started; MA, March; AP, April; MY, May; JN, June; JL, July; AU, August; SE, September; OC, October; NO, November.

Taken into account these results, we selected 1,411 fishing operation (47.54% of observed sets) operated using LLALB, LLHB, and LLAM from May to November, which present the 93% of total dolphinfish bycatches.

### 3.3. Partial LR Models

We adjusted the probability that a fishing operation present a CPUE value higher than the average CPUE for this boat stratum. We analysed three boat strata: LLALB, LLAM, and LLHB, from June to November along all the study period.

**Figure 7:**
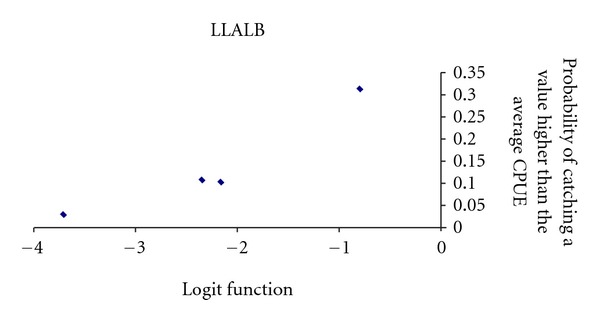
Probability of obtaining a CPUEw of dolphinfish in a LLALB set higher than the average CPUEw for LLALB.

For LLALB boat stratum (Figure 7), we obtained a statistically significant logistic model with the variables (in order of Wald-value): Moon effect (positive relation), and Diurnal setting (positive relation). The model's goodness of fit was significant according to the Omnibus test (Omnibus test = 18.775, *df* = 2, *P* < 0.001; Hosmer and Lemeshow test = 0.501, *df* = 2, *P* = 0.778). *R* ^2^-Nagalkerke = 0.14, and its discrimination capacity was outstanding (AUC = 0.7).

The logit function (*y*) from logistic regression was

(2) $$\begin{array}{c} y=-2.164+\text{MOON}\{\begin{array}{c} \text{NOT}=0\\ \text{YES}=1.37 \end{array}+\text{DN}\{\begin{array}{c} \text{NOT}=-1.549\\ \text{YES}=0. \end{array} \end{array}$$

Key words: DN, Diurnal or nocturnal setting; MOON, Moon effect.

**Figure 8:**
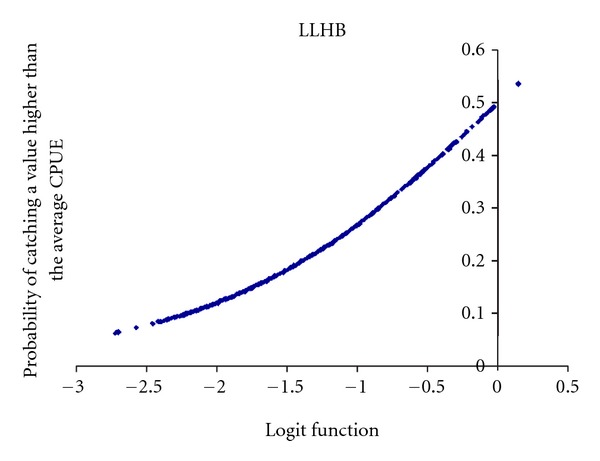
Probability of obtaining a CPUEw of dolphinfish in a LLHB set higher than the average CPUEw for LLHB.

In the case of LLHB boat stratum (Figure 8), we obtained a statistically significant logistic model with the variables (in order of Wald-value): Moon effect (positive relation) and Sea Surface Temperature where the setting started (negative relation). The model's goodness of fit was significant according to the Omnibus test (Omnibus test = 48.822, *df* = 2, *P* < 0.001; Hosmer and Lemeshow test = 28.377, *df* = 8, *P* < 0.001). *R* ^2^-Nagalkerke = 0.078, and its discrimination capacity was outstanding (AUC = 0.668).

The logit function (*y*) from logistic regression was

(3) $$y=2.952+\text{MOON}\{\begin{array}{c} \text{NOT}=0\\ \text{YES}=0.406 \end{array}+{\text{SSTSS}}^{*}-0.193.$$

Key words: MOON, Moon effect; SSTSS, Sea Surface Temperature where the setting started.

**Figure 9:**
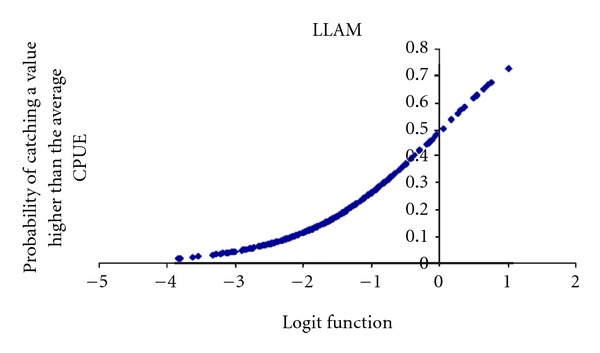
Probability of obtaining a CPUEw of dolphinfish in a LLAM set higher than the average CPUEw for LLAM.

In the particular case of LLAM boat stratum (Figure 9), we obtained a statistically significant logistic model with the variables (in order of Wald-value): Longitude where the setting started (negative relation) and Sea Surface Temperature where the setting started (negative relation). The model's goodness of fit was significant according to the Omnibus test (Omnibus test = 35.479, *df* = 2, *P* < 0.001; Hosmer and Lemeshow test = 18.233, *df* = 8, *P* = 0.02). *R* ^2^-Nagalkerke = 0.185, and its discrimination capacity was outstanding (AUC = 0.765).

The logit function (*y*) from logistic regression was

(4) $$y=9.417+{\text{LONGSS}}^{*}-0.438+{\text{SSTSS}}^{*}-0.416.$$

Key words: LONGSS, Longitude where the setting started; SSTSS, Sea Surface Temperature where the setting started.

## 4. Discussion

Our results indicate that the impact of the pelagic and semipelagic longline on the dolphinfish population is relatively low (1.083 fishes per 1000 hooks), in contrast with the higher effect on the target species population. LLALB is the gear with the highest effect on dolphinfish populations (CPUEn = 3.7 fishes per 1000 hooks) and has a remarkable incidence on juveniles. We suggest that this gear could be interacting with other artisanal fisheries targeting dolphinfish around Mallorca Island (Lleonart et al., [5]). In this sense, it is interesting to note the low catch rates of dolphinfish bycaught by LLALB around this area, in contrast with highest CPUEs in areas south-east of Menorca Island and the Ebro Delta continental shelf. LLAM (CPUEn = 1.2 fishes per 1000 hooks) and LLHB (CPUEn = 0.9 fishes per 1000 hooks) follow to LLALB in the catch rate ranking. LLAM and LLHB show a more homogeneous geographical distribution of their catch rates and also lower catch rates by set that LLALB.

In our study, LLJAP, LLSP, and LLPB had the lowest catch ratios of dolphinfish. Differences in bycatch rates can be attributed to differences both in selectivity between gears and fishing strategy. In this sense, LLALB operates with smaller hooks and bait, affecting mainly juvenile fraction of dolphinfish population. Interestingly, LLJAP, LLSP, and LLPB catch the largest dolphinfish and mainly affect the adult fraction of the population. We suggest that there is a relation between the fishing depth and the length of the fishes caught by the longline, and also between the size of the hooks and the mean length of the dolphinfish caught. Therefore, the largest captures correspond to LLJAP (105 cm) that operates at 250 f in deep and with the large hooks, the LLSP follows LLJAP in mean length of the dolphinfish caught (96.2 cm) and operates at 200 f in deep and also with large hooks. Finally, LLPB operates between 50 f and 250 f in deep and obtained a mean length of 79 cm for dolphinfish caught. Due to the fact that LLSP had the shortest temporal series (2007–2010) and that sampling coverage was lower, more attention should be paid to this gear in the future in order to determine its real impact on dolphinfish.

The Spanish longline fishery captures of dolphinfish in our study was 14,490 fishes per year (24.2 t), which is lower than that reported for artisanal fisheries by other authors in the Mediterranean, 63 t in Mallorca [5] and 377.4 t in Sicily [6], but is important in terms of assessment and management purposes.

The technical characteristic of the fishery and seasonality factors plays an important part in the absence or presence of dolphinfish bycatch in the different boat strata, gear type, and season. Moreover, as discussed previously, we also noted differences in size and weight of dolphinfish caught by the different gear types. In this context, our results suggest that longline should not be considered a simple boat stratum and gear type. In addition, our results indicate a seasonal increase in the catch ratios from June to November, which is in agreement with dolphinfish seasonal migrations in the Mediterranean [6].

Our results about particular LR models (per boat strata) indicate that environment factors could be the most important factors affecting CPUEw. We found a negative relationship between CPUEw per LLAM and SSTSS (see Figure 9). This particular relation could be explained for the oceanographic context in which this fishery takes place. Thus, in the Western Mediterranean Sea during summer upwelling frequently occurs near to the coast [22]. The upwelling increases the nutrients and reduces the SST. Many pelagic fish use these productive upwellings as feeding areas. Dolphinfish could be more abundant in these feeding areas, thus increasing their catchability and consequently the CPUEw. For this reason, the negative relationship between LLAM, CPUEw, and the LONGSS could be related with this trend. The relationship between dolphinfish catches and ocean temperature has been cited in many studies (e.g., [23, 24]). The majority of these studies suggested positive correlations between dolphinfish catch ratios and sea surface temperatures (SST). However, the SST in these studies was considered as a global variable in the study area and not a particular value of each fishing operation.

Lunar phase has been frequently used as an explanatory variable affecting catch rates of dolphinfish [25]. Generally the lunar phases from new moon to the first quarter increase the catch ratios of this species. However, our data indicated that, the more brilliant a nocturnal set was, the more abundant was the bycatch of dolphinfish. Nevertheless, we found that the highest catch ratios occur in those fishing operations carried out in diurnal hours. For this reason, we suggest that our results are more in relation with the gravitational effect related with the moon phase than to the light effect of moon phases.

## Figures and Tables

**Table 1:** Six boats strata and main characteristics of each stratum. Key words: LLALB, Drifting surface longliners targeting albacore; LLHB, Traditional longliners targeting swordfish; LLAM, American longliners targeting swordfish; LLJAP, Drifting surface longliners targeting bluefin tuna; LLSP, Drifting semipelagic longliners targeting swordfish; LLPB, Demersal longliners targeting swordfish.

Target species	Pelagic drifting	Semipelagic and demersal
Albacore	LLALB	
Bluefin Tuna	LLJAP	
Swordfish	LLHB, LLAM	LLSP, LLPB

**Table 2:** Factors and explanatory variables used in the general logistic regression model.

Factors	Variables	Variables type	Abbreviation
Dependent variable	Absence/presence Coryphaena bycatches per set	Binary	CO

Technical characteristics of the fishery	Number of hooks	Quantitative	NH
	Distance between both extremes of the longline	Quantitative	DL
	Diurnal or nocturnal setting	Binary	DN
	Setting hours	Categorical	
	Drifting surface longliners targeting bluefin tuna	Binary	LLJAP
	Traditional longliners targeting swordfish	Binary	LLHB
	American longliners targeting swordfish	Binary	LLAM
	Drifting surface longliners targeting albacore	Binary	LLALB
	Drifting semipelagic longliners targeting swordfish	Binary	LLSP
	Demersal longliners targeting swordfish	Binary	LLPB

Geographical location	Latitude where the setting started	Quantitative	LATSS
	Longitude where the setting started	Quantitative	LONGSS
	Latitude where the setting finished	Quantitative	LATFS
	Longitude where the setting finished	Quantitative	LONGFS
	Sets over continental shelf	Binary	SCS

Seasonality (phenology)	January	Binary	JA
	February	Binary	F
	March	Binary	MR
	April	Binary	AP
	May	Binary	MY
	June	Binary	JN
	July	Binary	JL
	August	Binary	AU
	September	Binary	S
	October	Binary	O
	November	Binary	N
	December	Binary	D

**Table 3:** Factors and explanatory variables used in the partial logistic regression models. Abbreviations: CPUE, Capture per unit of effort; SSTSS, Sea surface temperature at starting set; SSTFS, Sea surface temperature in the final of the set.

Factors	Variables	Variables types	Abbreviation
Dependent variable	the probability of a fishing operation presents a CPUE value higher than the average CPUE for this boat stratum	Binary	COcpue

Technical characteristics of the fishery	Distance between both extremes of the longline	Quantitative	DL
	Diurnal or nocturnal setting	Binary	DN

Geographical location	Latitude where the setting started	Quantitative	LATSS
	Longitude where the setting started	Quantitative	LONGSS
	Latitude where the setting finished	Quantitative	LATFS
	Longitude where the setting finished	Quantitative	LONGFS
	Sets over continental shelf	Binary	SCS

Environment	Sea Surface Temperature where the setting started	Quantitative	SSTSS
	Sea Surface Temperature where the setting finished	Binary	SSTFS
	Mean of Sea Surface Temperature between SSTSS and SSTFS	Binary	MR
	Absolute variation between SSTSS and SSTFS	Binary	AP
	Moon effect	Binary	MO

**Table 4:** Annual nominal CPUEn per gear type.

	2000	2001	2002	2003	2004	2005	2006	2007	2008	2009	2010
LLALB	0.054	blank	blank	blank	blank	blank	9.996	0.376	5.024	0.061	1.631
LLHB	0.894	1.613	0.146	0.811	1.693	1.281	1.093	0.657	0.122	0.265	0.094
LLAM	blank	blank	blank	2.286	1.085	0.000	1.261	1.150	0.411	0.000	blank
LLSP	blank	blank	blank	blank	blank	blank	blank	0.123	0.116	0.003	0.028
LLJAP	0.000	0.162	0.000	0.000	0.000	0.000	0.000	0.147	0.000	0.000	0.000
LLPB	0.000	0.000	0.000	0.000	0.000	blank	0.032	0.037	0.322	0.127	0.126

**Table 5:** Annual nominal CPUEw per gear type.

	2000	2001	2002	2003	2004	2005	2006	2007	2008	2009	2010
LLALB	0.041	blank	blank	blank	blank	blank	7.687	0.289	3.864	0.047	1.254
LLHB	2.423	4.372	0.397	2.199	4.590	3.474	2.962	1.781	0.330	0.718	0.256
LLAM	blank	blank	blank	2.439	1.157	0.000	1.346	1.227	0.439	0.000	blank
LLSP	blank	blank	blank	blank	blank	blank	blank	0.554	0.522	0.015	0.124
LLJAP	0.000	1.362	0.000	0.000	0.000	0.000	0.000	1.241	0.000	0.000	0.000
LLPB	0.000	0.000	0.000	0.000	0.000	blank	0.360	0.413	3.589	1.421	1.407
